# Vibrational Predissociation Spectra of C_2_N^−^ and C_3_N^−^: Bending and Stretching Vibrations

**DOI:** 10.1002/cphc.202300262

**Published:** 2023-07-06

**Authors:** Franziska Dahlmann, Dennis F. Dinu, Pavol Jusko, Christine Lochmann, Thomas Gstir, Aravindh N. Marimuthu, Klaus R. Liedl, Sandra Brünken, Roland Wester

**Affiliations:** ^1^ Institut für Ionenphysik und Angewandte Physik Universität Innsbruck Technikerstraße 25 6020 Innsbruck Austria; ^2^ Department of General Inorganic and Theoretical Chemistry Universität Innsbruck Innrain 80/82 6020 Innsbruck Austria; ^3^ Max Planck Institute for Extraterrestrial Physics Gießenbachstraße 1 85748 Garching Germany; ^4^ Radboud University Institute for Molecules and Materials FELIX Laboratory Toernooiveld 7 6525ED Nijmegen, the Netherlands

## Abstract

We present infrared predissociation spectra of C_2_N^−^(H_2_) and C _3_N^−^(H_2_) in the 300–1850 cm^−1^ range. Measurements were performed using the FELion cryogenic ion trap end user station at the Free Electron Lasers for Infrared eXperiments (FELIX) laboratory. For C_2_N^−^(H_2_), we detected the CCN bending and CC−N stretching vibrations. For the C_3_N^−^(H_2_) system, we detected the CCN bending, the CC−CN stretching, and multiple overtones and/or combination bands. The assignment and interpretation of the presented experimental spectra is validated by calculations of anharmonic spectra within the vibrational configuration interaction (VCI) approach, based on potential energy surfaces calculated at explicitly correlated coupled cluster theory (CCSD(T)‐F12/cc‐pVTZ−F12). The H_2_ tag acts as an innocent spectator, not significantly affecting the C_2,3_N^−^ bending and stretching mode positions. The recorded infrared predissociation spectra can thus be used as a proxy for the vibrational spectra of the bare anions.

## Graphical abstract

**Infrared predissociation spectra** of C_2_N^−^(H_2_) and C_3_N^−^(H_2_) is reported using a cryogenic ion trap at the FELIX Laboratory, thus allowing the detection of low‐lying bending and stretching vibrations of both anions simultaneously. Vibrational configuration interaction calculations validate the assignment. The recorded spectra can be used as a proxy for the vibrational spectra of the bare anions since the tag does not significantly affect the mode position.

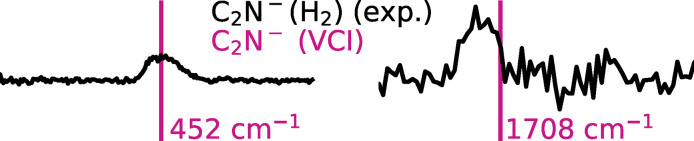

## Introduction

The linear carbon chains of the type C_*n*_N^−^ attracted much interest in theoretical and experimental studies over recent years,^[1]^ encouraged by the discoveries of CN^−^, C_3_N^−^, C_5_N^−^, and very recently C_7_N^−^ in the circumstellar envelope of the late‐type star IRC+10216 with high‐resolution radio spectroscopy in combination with laboratory data or high‐level ab‐initio calculations.[^2^, ^3^, ^4^, ^5^, ^6^] C_3_N^−^ and C_5_N^−^ were recently also detected in the cold molecular cloud TMC‐1,^[7]^ whereas only an upper limit was found for CN^−^ in this source.^[3]^ The observed abundances hint towards efficient formation pathways via reaction of nitrogen atoms with longer carbon chain anions $C_{n}^{-}$ rather than via direct radiative attachment. In contrast, no member of the C_2*n*_N^−^ group has been detected in space, even though they are predicted to play a role in the chemical pathways of the interstellar medium (ISM).[^8^, ^9^] This is likely due to their open shell electronic ground states (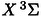 ) leading to increased reactivity but also larger rotational partition functions, hampering not only radio‐astronomical detection but also their laboratory spectroscopic characterization.

The neutral C_2_N (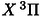 ) radical has been characterized in a variety of electronic states by means of electronic absorption and vibrational emission spectroscopy,[^10^, ^11^, ^12^] laser‐induced fluorescence spectroscopy^[13]^ and infrared (IR) spectroscopy and far‐infrared laser magnetic resonance for selected bands.[^14^, ^15^] Through matrix isolation infrared spectroscopy,[^16^, ^17^, ^18^] the C_2_N radical has been observed as a byproduct in the degradation of various carbon‐nitrogen species, such as acetonitrile (CH_3_CN),[^16^, ^18^] or cyanogen (NCCN).^[17]^ C_2_N is also well studied from a theoretical point of view.[^19^, ^20^] For the C_*n*_N^−^ anion, the cases n=1-7 have been investigated at several levels of theory by Pascoli et al.^[21]^ and rotational state‐changing dynamics for C_2_N^−^ have been reported by Franz et al.^[22]^ Experimentally, high resolution photoelectron spectra for C_2_N^−^, C_4_N^−^, and C_6_N^−^ have been detected by slow electron velocity map imaging.^[23]^ For C_2_N^−^ the authors assigned the fundamental bending vibration. Up to now, further spectroscopic signatures were only theoretically predicted by Rocha and Linnartz.^[24]^ They performed extensive anharmonic calculations for C_2_N^−^ and CNC^−^ using vibrational perturbation theory of second order (VPT2) to solve the three‐atom rovibrational Hamiltonian by discrete variable representation (DVR3D).^[25]^ As the C_2_N^−^ is a triplet (or biradical) in its electronic ground state,^[24]^ its high reactivity poses experimental challenges for its production. This has led to a lack of gas‐phase vibrational measurements, e. g., using infrared predissociation (IRPD) spectroscopy, so far. To our knowledge, no previous measurements of the vibrational predissociation spectra of tagged C_2_N^−^ anions exist.

Odd‐numbered C_*n*_N species are less reactive than the even‐numbered species due to their closed shell ^1^Σ electronic ground states,[^26^, ^27^] and are thus easier to isolate in experiments. Vibrational bands of C_3_N^−^ were identified using krypton‐, argon‐ and neon‐matrix IR isolation spectroscopy, supported by anharmonic VPT2 frequency calculations based on cubic force fields at the coupled cluster level of theory by Kołos et al..^[28]^ This leads to the assignment of the C_3_N^−^ absorption feature around 2180 cm^−1^ to be the C−N stretching.[^29^, ^30^] The C−CCN stretching band at ∼1944 cm^−1^ was also detected in a krypton, and argon matrix.^[28]^ Fourier transform microwave spectroscopy was used to measure the rotational spectra of C_3_N^−^ in the vibrational ground state with high spectral resolution.[^2^, ^31^] Yen et al.^[32]^ extracted the vibrational frequencies of the cis and trans bending modes of C_3_N^−^ with photoelectron spectroscopy using slow electron velocity‐map imaging (SEVI). Cryogenic IRPD spectra of C_3_N^−^ were obtained by Stanca‐Kaposta et al.^[33]^ in the spectral range of the C−N and C−CCN stretching bond (1850–2400 cm^−1^) with D_2_ as messenger tag. The bands were assigned based on scaled harmonic frequencies at CCSD(T) level of theory. As far as we know, there are no experimental records available for the vibrational predissociation spectrum of C_3_N^−^ using any tagging agent at frequencies lower than 1850 cm^−1^.

The advent of cryogenic multipole radio‐frequency ion traps and buffer‐gas cooling has contributed to investigate weakly‐bound ionic complexes by IRPD spectroscopy.[^34^, ^35^, ^36^, ^37^, ^38^, ^39^] Here, we extend our studies on CI^−^(H_2_)[^40^, ^41^] and CI^−^(H_2_)^[42]^ and present the vibrational spectra of dihydrogen polycyanides C_*n*_N^−^(H_2_), n=2 and 3 using the same experimental approach, i. e., cryogenic infrared predissociation spectroscopy. Two stable conformers (C_2_N^−^(H_2_) or (H_2_)C_2_N^−^ and C_3_N^−^(H_2_) or (H_2_)C_3_N^−^, respectively) can be formed. In contrast to CN^−^(H_2_), where we studied the low‐lying intermonomer vibrations, we focus here on the vibrational modes of the C_2_N^−^ and C_3_N^−^ anionic core. To aid the assignment and interpretation of the experimental spectra, we provide anharmonic frequency calculations suited for calculation of these species. Additionally, previous theoretical studies and similar experimental studies are used for comparison. For C_3_N^−^ with H_2_ the potential energy surface (PES) has been developed and the rovibrational bound states have been calculated by Lara‐Moreno et al.^[43]^ Rocha and Linnartz^[24]^ and Kołos et al.^[28]^ have shown that accurate prediction of the vibrational structure of the C_2_N^−^ and C_3_N^−^ species can be achieved based on Taylor series expansions of the PES. It was shown that vibrational perturbation theory of second order (VPT2) is a viable way to calculate the spectroscopic features of these species[^24^, ^28^]. In the present work, we extend and support these theoretical predictions with an alternative approach. We calculate multi‐mode expansions of the PES at explicitly correlated coupled cluster theory, and perform anharmonic calculations using a variational approach, i. e., vibrational configuration interaction (VCI).^[44]^ In contrast to the variational DVR3D approach^[25]^ used by Rocha and Linnartz,^[24]^ we are not limited to three‐atomic systems, making it possible to calculate both C_2_N^−^ and C_3_N^−^ with the same accuracy. Even though substancial differences between the vibrational spectra of the *para* and *ortho* nuclear spin isomers have to be expected, we focus on the intramolecular bending and stretching vibrations, not on the intermolecular vibrations including the H_2_ tag. The reason is that the theoretical treatment of this system with an accurate quantum approach is presently too complex due to the longer chain length.^[42]^

## Results and Discussion

**Figure 1:**
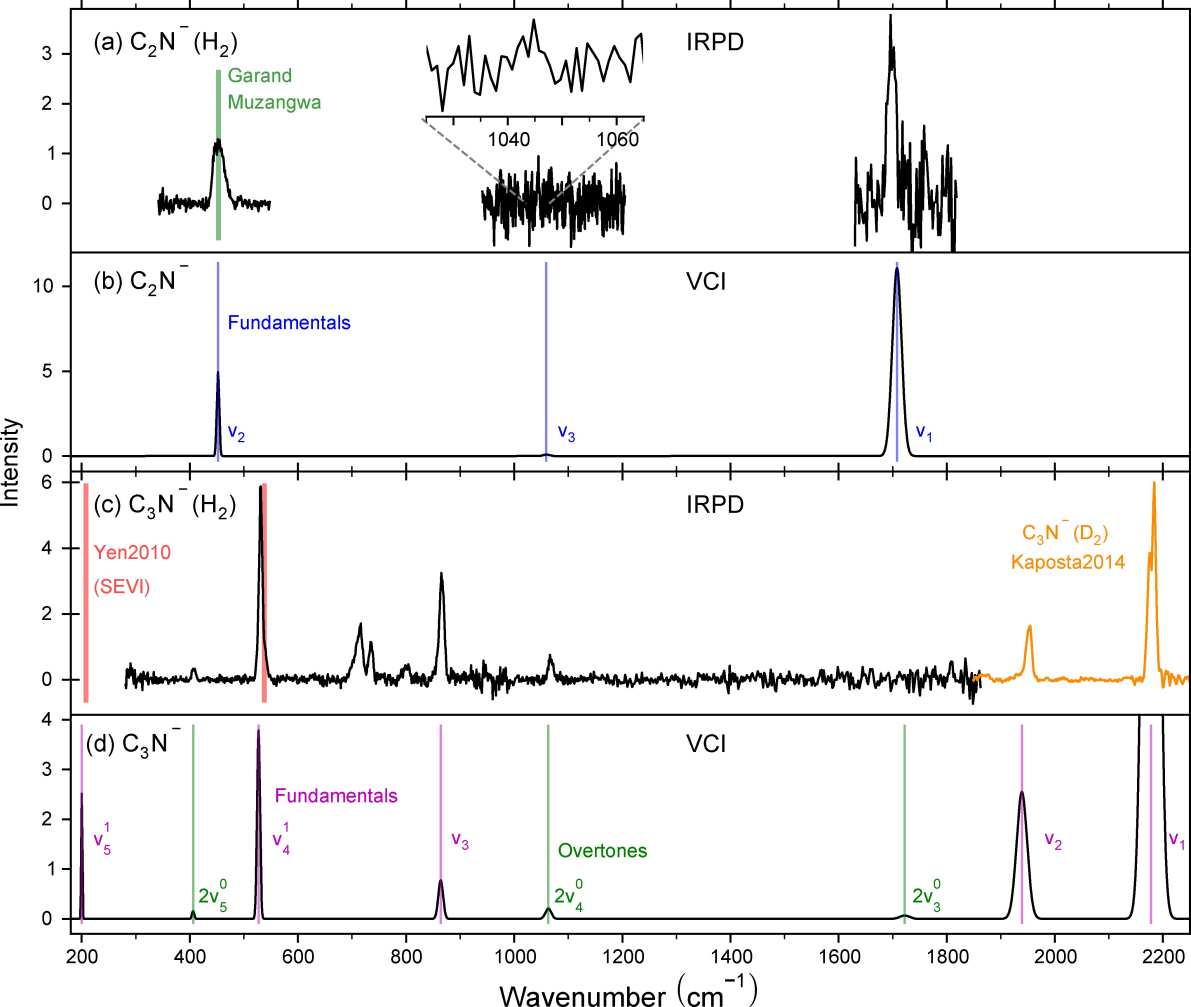
a) Experimental vibrational infrared predissociation spectra of C_2_N^−^(H_2_) recorded at 15 K trap temperature. The green line shows the experimental estimate of Rocha and Linnartz,^[24]^ derived from slow electron velocity map imaging spectroscopy of C_2_N^−[23]^ and newer spectroscopy of neutral CCN.^[45]^ b) The black trace shows the predicted frequencies and band intensities for bare C_2_N^−^ from our VCI calculations, folded with the line width of the FELIX FEL‐2 laser. Blue lines show the frequencies taken from Ref. [24]. c) Experimental vibrational infrared predissociation spectra of C_3_N^−^(H_2_) recorded at 15 K. IRPD spectra of C_3_N^−^(D_2_) from Stanca‐Kaposta et al. (intensity not to scale) are shown in orange.^[33]^ Red lines are frequencies obtained by slow electron velocity map imaging^[32]^ d) The black trace shows the predicted frequencies and band intensities for bare C_3_N^−^ from our VCI calculations, folded with the line width of the FELIX FEL‐2 laser. The violet and green lines mark the fundamental and overtone bands, respectively.

**C_2_N^−^**: The single photon IRPD spectrum of C_2_N^−^ tagged with H_2_ is shown in the top panel of Figure 1, which reveals two clearly visible vibrational bands. The second panel shows calculated vibrational band positions and intensities of bare C_2_N^−^ for comparison. In Table 1 our values are listed together with the frequencies from Rocha and Linnartz,^[24]^ denoted VAR (an extended Table including harmonic calculations and combination bands is found in Table S1).

**Table 1:** Experimental frequencies of C_2_N^−^(H_2_) and anharmonic frequencies of the bare C_2_N^−^ anion. Positions are given in cm^−1^, intensities in km⋅mol^−1^ (theo.) and relative intensity (exp.).

Mode	State	IRPD^*a*^	VCI ^*b*^	VAR^*c*^
		freq./Int.	freq./Int.	
$\nu_{\mathrm{CC}-N}$	*ν*_1_ (Σ^+^)	1698(1)/3.6	1708/236.5	1696.5
$\nu_{C-\mathrm{CN}}$	*ν*_3_ (Σ^+^)	–	1058/1.3	1045.8
$\delta_{\mathrm{CCN}}$	$\nu_{2}^{1}$ (Π)	454(1)/1.2	452/28.4	452.1
2$\nu_{\mathrm{CC}-N}$	2*ν*_1_ (Σ^+^)		3393/73.4	
2$\nu_{C-\mathrm{CN}}$	2*ν*_3_ (Σ^+^)		2100/0.5	
2$\delta_{\mathrm{CCN}}$	2$\nu_{2}^{0}$ (Σ^+^)		893/3.5	

^*a*^ This work: Infrared predissociation on C_2_N^−^(H_2_), ^*b*^ This work: Vibrational configuration interaction (VCI) calculations on a multi‐mode PES with up to 3‐mode couplings at the CCSD(T)‐F12 level of theory, ^*c*^ Ref. [24]: Variational (VAR) solution of the three‐atom Hamiltonian on a composite quartic force field at CCSD(T) level of theory.

The strongest feature is detected at 454(1) cm^−1^. This value is in good agreement with the anharmonic frequency of 452 cm^−1^ obtained from our VCI calculations based on a multi‐mode PES with up to 3‐mode coupling at the CCSD(T)‐F12/cc‐pVTZ−F12 level of theory. Assigning this vibrational band to the $\delta_{\mathrm{CCN}}$ (*ν*_2_) bending vibration is additionally supported by the accurate state‐of‐the‐art rovibrational quantum chemical calculations from Rocha and Linnartz,^[24]^ determining *ν*_2_ to be at 452.1 cm^−1^ using variational DVR3D calculations.

The bending vibration $\delta_{\mathrm{CCN}}$ (*ν*_2_) for C_2_N^−^ was experimentally assigned by Garand et al. using high‐resolution photoelectron spectroscopy by slow electron velocity map imaging (SEVI). They assigned a value of 432 cm^−1^ to the bending fundamental.^[23]^ A different experimental estimate for the C_2_N^−^ bending vibration, at 452.9±2.9 cm^−1^, was derived by Rocha and Linnartz^[24]^ (shown as green vertical line in Figure 1). To derive this value, the photoelectron spectroscopic data of Garand et al.^[23]^ was combined with the revisited energy splitting in neutral CCN reported by Muzangwa et al..^[45]^ This revised SEVI value is in good agreement with our IRPD measurement, indicating that the H_2_ tag has only a minor impact on the band position.

A second strong feature was detected in the present work at 1698(1) cm^−1^. The anharmonic frequencies from our VCI calculations predict the $\nu_{\mathrm{CC}-N}$ stretch vibration (*ν*_1_) at 1708 cm^−1^, slightly higher than the calculations by Rocha and Linnartz,^[24]^ supporting the assignment to the C−N stretch vibration. This vibration is predicted to be shifted by less than 2 cm^−1^ by the H_2_ tag based on harmonic calculations (see Table S4), supporting the argument of the innocent spectator in this system.

The deviations in the calculated frequencies between the present VCI calculations and the DVR3D results by Rocha et al. are about 10 cm^−1^ for the stretching vibrations. These deviations are most likely due to the different choice of electronic structure theory and in the different representation of the PES as a quartic force field or a multi‐mode expansion. Our harmonic calculations of hydrogen tagged C_2_N^−^(H_2_) calculated at the CCSD(T)‐F12/cc‐pVTZ−F12 level of theory show that the H_2_ tag does influence the bending vibrations only marginally, i. e., inducing shifts of about 3 cm^−1^ and about 1 cm^−1^ when the nitrogen‐side tagged or the carbon‐side are tagged, respectively (see Table S4). For this reason we compare with accurate anharmonic calculations of bare anions, instead of explicitly including the H_2_ tag at a lower computational accuracy.

The $\nu_{C-\mathrm{CN}}$ stretch vibration (*ν*_3_) is predicted to be at at 1058 cm^−1^ by our VCI calculations and at 1045.8 cm^−1^ by Rocha and Linnartz.^[24]^ We did not detect a band in the range from 933 to 1203 cm^−1^. Using the signal‐to‐noise ratio from our experiment for the bending vibration *ν*_2_, we can give an upper limit for the *ν*_3_ intesity. The noise level was approximately three times higher in the predicted region of the *ν*_3_ band. According to the VCI predictions, the intensity of *ν*_3_ should be about 20 times smaller than the bending one, resulting in an expected signal‐to‐noise ratio of about 0.3. We did not observe such a weak feature (see also insert in Figure 1, suggesting that the *ν*_3_ vibration is indeed weak. Based on the agreement with the anharmonic calculations for *ν*_2_ and *ν*_1_, the tag is unlikely to shift the band position significantly. The dipole moment for this transition may be weak, resulting in no detection.

**C_3_N^−^**: The third panel of Figure 1 shows the IRPD spectrum of C_3_N^−^(H_2_). Several vibrational bands are observed in the covered range of 350 to 1850 cm^−1^. The strongest feature is at 530(1) cm^−1^. Yen et al.^[32]^ extracted the $\delta_{\mathrm{CCN}}$ and $\delta_{\mathrm{CCC}}$ bending modes from the SEVI spectra to be at 538±8 and 208±8 cm^−1^, respectively. Our VCI calculation, shown in the fourth panel of Figure 1, supports this assignment with $\nu_{4}^{1}$ at 527 cm^−1^ and $\nu_{5}^{1}$ at 200 cm^−1^. Thus, we assign the IRPD band at 530 cm^−1^ to the $\delta_{\mathrm{CCN}}$ bending mode (see Table 2). Due to the strength of the fundamental band, also the 2$\delta_{\mathrm{CCN}}$ overtone is detected at 1068(1) cm^−1^, supported by our VCI calculation of $2\nu_{4}^{0}$ predicted at 1063 cm^−1^. An extended Table with additional calculated values is found in Table S2.

**Table 2:** Experimental frequencies of C_3_N^−^(H_2_) and anharmonic frequencies of the bare C_3_N^−^ anion. Positions are given in cm^−1^, intensities in km⋅mol^−1^ (theo.) and relative intensity (exp.).

Mode	State	IRPD	VCI^*c*^	Anharm.^*d*^
		freq./Int.	freq./Int.	freq./Int.
$\nu_{\mathrm{CCC}-N}$	*ν*_1_ (Σ^+^)	2180^*a*^	2178/804	2182.3/476
$\nu_{C-\mathrm{CCN}}$	*ν*_2_ (Σ^+^)	1952^*a*^	1939/62	1940.9/46
$\nu_{\mathrm{CC}-\mathrm{CN}}$	*ν*_3_ (Σ^+^)	866(1)/3.1^*b*^	864/8	866.7/10
$\delta_{\mathrm{CCN}}$	*ν*_4_^1^ (Π)	530(1)/5.8^*b*^	527/25	
$\delta_{\mathrm{CCC}}$	*ν*_5_^1^ (Π)		200/6	
2$\nu_{\mathrm{CCC}-N}$	2*ν*_1_ (Σ^+^)		4336/1	
2$\nu_{C-\mathrm{CCN}}$	2*ν*_2_ (Σ^+^)		3865/2	
2$\nu_{\mathrm{CC}-\mathrm{CN}}$	2*ν*_3_ (Σ^+^)		1722/2	
2$\delta_{\mathrm{CCN}}$	2*ν*_4_^0^ (Σ^+^)	1068(1)/0.6^*b*^	1063/3	
2$\delta_{\mathrm{CCC}}$	2*ν*_5_^0^ (Σ^+^)	407(1)/0.3^*b*^	406/1	

^*a*^ Ref. [33]: (IRPD) of C_3_N^−^(D_2_), ^*b*^ This work: IRPD of C_3_N^−^(H_2_), ^*c*^ This work: Vibrational configuration interaction (VCI) based on a multi‐mode PES with up to 3‐mode couplings at CCSD(T)‐F12 level of theory. ^*d*^ Ref. [28]: Vibrational perturbation theory (VPT2) based on a cubic force field at CCSD(T) level of theory.

The second strongest band is at 866(1) cm^−1^, which matches the $\nu_{\mathrm{CC}-\mathrm{CN}}$ stretching mode (*ν*_3_) predicted from VPT2 calculations by Kołos et al.^[28]^ Our VCI calculations substantiate this assignment. In general, for the bands *ν*_1_, *ν*_2_, and *ν*_3_, we observe agreement between the VCI calculations and the VPT2 calculations by Kołos et al.^[28]^ of less than 4 cm^−1^. Note, however, that the calculated VCI intensities are only qualitatively reproducing the experimentally detected intensities.

The lowest‐frequency feature at 407(1) cm^−1^ is most likely the second overtone of the cis $\delta_{\mathrm{CCN}}$ bending mode (2*ν*_5_). The assignment is supported by our VCI calculations with position and intensity, predicting 2*ν*_5_ at 410(1) cm^−1^. This assignment is based on the fact that the cis bending *ν*_4_ is the strongest one detected, allowing the assumption that also the $\delta_{\mathrm{CCC}}$ trans bending (*ν*_5_) will be strong. The *ν*_5_ fundamental lies at about 150 cm^−1^ below the FEL‐2 limit and was thus not covered in this study.

Stanca‐Kaposta et al.^[33]^ measured the C_3_N^−^ IRPD spectrum from 1850 cm^−1^ upwards and detected the $\nu_{\mathrm{CCC}-N}$ (*ν*_1_) and $\nu_{C-\mathrm{CCN}}$ (*ν*_2_) stretching vibrations at 2180 and 1952 cm^−1^, respectively. This spectrum is shown as an orange line in Figure 1(c). Please note that the intensities cannot be compared to those presented in our study. They were able to assign the experimental band position and intensities with harmonic frequency calculations at CCSD(T) level of theory. Our anharmonic VCI calculations support this assignment. All calculated C_3_N^−^ values can be found in the extended Table S2.

After identifying all C_3_N^−^ bare ion vibrational modes in the spectrum, three vibrational modes remain unassigned, at 713(1), 735(1) and 800(1) cm^−1^. The feature at 713(1) cm^−1^ deviates from a Gaussian shape observed in the other features. When combined with the 735(1) cm^−1^ band, it fits the shape predicted by accurate quantum approach theory for the second overtone of the hindered rotation of H_2_ in the CN^−^(H_2_) system.^[42]^ The first two features might thus include an H_2_ contribution. The corresponding overtones were detected at 773 cm^−1^ for CN^−^(H_2_)^[42]^ and at 755 cm^−1^ for CI^−^(H_2_).^[41]^ In this case, the last band detected at 800(1) cm^−1^ can tentatively be assigned to the hindered rotation of the H_2_ in combination with an H_2_ intermolecular stretching vibration, similar to CI^−^(H_2_). Alternatively, the peak at 735(1) cm^−1^ could consist or contain the combination of *ν*_4_+*ν*_5_ predicted to be at 738 cm^−1^, even though the calculation predicts a vanishing intensity (see Table S2).

The characteristic P, Q, and R‐substructure, seen in the vibrational bands at high temperatures, collapse at low temperatures, as previously demonstrated by Jusko et al.^[37]^ In the *ν*_1_ and *ν*_2_ stretching band of the D_2_ tagged system, the P and R branches have been assigned to rotationally excited C_3_N^−^(D_2_).^[33]^ Stanca‐Kaposta et al. concluded a mean rotational temperature of 75 K while maintaining a trap temperature of 15 K. It is known that the rotational temperature of trapped ions often does not fully thermalize.^[46]^ Simpson et al.^[47]^ used a similar trap to the one we employ here and found the best fit for the rotational temperature at 25 K. This, combined with the absence of a double‐peak structure in our presented spectra indicates that the ions in our experiment likely have a rotational temperature in the order of 25 K.

In the previously studied system of C_3_N^−^(D_2_), the influence of the tag molecule has been discussed in detail.^[33]^ There, they find that the C‐side tagged complex is preferred by 84 cm^−1^. For C_3_N^−^(H_2_), we can confirm the preference of H_2_ for the C‐side tagging, where our calculations show an energy difference of 73 cm^−1^. For all the newly detected fundamental stretching and bending vibrations our calculations predict band shifts induced by the H_2_ tag of less than 4 cm^−1^ independent of the tagging side using harmonic calculations (see Table S4), supporting the argument of the innocent spectator in this system. Due to our laser bandwidth being larger than the predicted shift, we can not distinguish between the different tagging sides experimentally. Considering that Stanca‐Kaposta et al.^[33]^ noted a shift of less than 1 cm^−1^ for the system of C_3_N^−^(D_2_), we can conclude that the complexation of the tag, whether it is D_2_ or H_2_ has a negligible effect on the measured vibrational frequencies.

Although the single‐tagged anion is formed with highest abundance, multiple tags can be formed in small numbers in the cryogenic trap (single tagging efficiency for C_3_N^−^(H_2_) is ∼6.0 %, double tagging ∼2.5 %). For the double‐tagged system C_3_N^−^(H_2_)_2_, we note a 3 cm^−1^ blue‐shift for the *ν*_4_ bending vibration (see Figure S1). To determine the actual shift caused by the single tag, it is necessary to compare the spectrum of the ion‐molecule complex to that of the bare ion. Possible approaches to achieve this goal are spectroscopy by laser‐inhibition of cluster growth^[48]^ or the recently demonstrated leak‐out spectroscopy,^[49]^ both of which allow for tag‐free spectroscopy.

Stanca‐Kaposta et al. predicted that the dissociation energy for the D_2_‐loss channel is smaller than 350 cm^−1^. Our calculations on the CCSD(T)‐F12/cc‐pVTZ−F12 level of theory predict a dissociation energy of 138 and 65 cm^−1^ for (H_2_)C_3_N^−^ and C_3_N^−^(H_2_), respectively (see SI and Table S3). This implies that also the *ν*_5_, predicted at 200 cm^−1^ should be detectable with a suitable laser system.

## Conclusion

Here, we present and discuss far‐infrared and mid‐infrared predisscociation vibrational spectra of the weakly bound complexes C_2_N^−^(H_2_) and C_3_N^−^(H_2_) in the region of their low‐lying stretching and bending modes of the bare anions and their intermonomer modes (300–1850 cm^−1^). By using a cryogenic ion trap instrument coupled to the widely tunable free‐electron lasers at the FELIX laboratory, we observed for C_2_N^−^(H_2_) the $\nu_{\mathrm{CC}-N}$ stretching vibration for the first time and can confirm the $\delta_{\mathrm{CCN}}$ bending vibration of C_2_N^−^ estimated by Ref. [24] with data from Garand et al.^[23]^ and Muzangwa et al..^[45]^ We could assign the bands with high‐level VCI calculations, supported by previous VAR calculations.^[24]^

In the system of C_3_H^−^(H_2_) we could access the low‐wavenumber region between 300–1850 cm^−1^ where the low‐lying bending and stretching bands are located. Combined with IRPD spectra on C_3_N^−^(D_2_) by Stanca‐Kaposta et al.^[33]^ starting 1850 cm^−1^ upwards, we can assign all fundamentals and several overtones. VCI calculations aid the assignment. The spectrum is dominated by the $\delta_{\mathrm{CCN}}$ bending vibration and the $\nu_{\mathrm{CC}-\mathrm{CN}}$ stretching vibration. As in the system of CN^−^(H_2_) we can tentatively assign one doublet feature to the second overtone of the bending vibration associated with the hindered rotation of H_2_.

The observation by Stanca Kaposta et al.^[33]^ of a small shift in the vibrational frequency of less than 1 cm^−1^ is confirmed by our calculations and leads us to conclude that the complexation of the tag has a negligible impact on the vibrational frequencies also in the low‐wavenumber regime. Thus the IRPD spectra obtained in the present work can be used as a substitute for the anions’ vibrational spectra in the tag's absence. In the future it will be interesting to study the influence of the H_2_ tag in more detail. This can serve as a benchmark for collisional excitation studies, see e. g.[^50^, ^51^]

## Methods

### Experimental

Experiments were performed at the FELion cryogenic ion trap beamline at the Free Electron Lasers for Infrared eXperiments (FELIX) Laboratory. The FELion instrument and its use for vibrational spectroscopic studies employing infrared‐predissociation action spectroscopy of rare‐gas tagged molecular ions have been described in detail previously.^[37]^ Here we only report the specific details related to the current investigation of the C_2_N^−^(H_2_) and C_3_N^−^(H_2_) systems.

C_2_N^−^ and C_3_N^−^ anions were produced by dissociative electron attachment to acetonitrile CH_3_CN and acrylonitrile C_3_H_3_N, respectively, in a Gerlich‐type ion storage source^[52]^ using electrons with energies around 70 eV . We want to note that the C_2_N^−^ yield was a factor of ∼100 smaller than the C_3_N^−^ yield. Ions with a mass‐to‐charge ratio m/z of either 38 (C_2_N) or 50 (C_3_N) were mass‐selected in a quadrupole mass filter and guided to the cryogenic 22 pole ion trap held at a fixed temperature of 15 K. At the beginning of the storage cycle, a mixture H_2_ : He of 1 : 2 was pulsed into the trap for 100 ms leading to cooling of the ions and the formation of C_2_N^−^(H_2_) or C_3_N^−^(H_2_) complexes. Normal H_2_ was used for the experiments described here. The complexes were stored between 1.5 and 2.6 s in the trap, where they were exposed to infrared radiation of the free‐electron laser FEL‐2 that delivered pulse energies varying from 20–35 mJ in the trap region at 10 Hz repetition rate with a macropulse duration of ∼10 μs. The laser was tuned in the region between 300–1850 cm^−1^, and the depletion of the number of complex ions due to single‐photon IRPD was recorded as a function of the frequency. The FEL‐2 was optimized for narrow bandwidth, reaching typical rms widths of about 0.5–0.6 % of the center value,^[37]^ or a FWHM of about 12 cm^−1^ at 1000 cm^−1^ in the present experiment. To account for varying laser pulse energy *E*, pulse numbers *n*, baseline drifts due to varying source conditions, and saturation effects, the signal is normalized prior to averaging using $I=\frac{-ln\left(S/B\right)}{n\cdot E}$ , with *S* the number of complexes as a function of laser frequency and *B* the number of baseline complexes. As the photon wavelength increases 5‐fold from 300 cm^−1^ to 1800 cm^−1^, the number of photons decreases 5‐fold assuming equivalent pulse energy. The reported experimental intensities are corrected to change in photon number as a function of wavelength.

### Computational

The C_*n*_N^−^ anion can be treated as a linear system^[21]^ in the $C_{\infty v}$ point group, with the electronic ground state being a singlet (^1^Σ) for the CN^−^ and C_3_N^−^ species and a triplet (^3^Σ) for the C_2_N^−^ species. Note that also non‐linear geometries and different electronic states were studied for C_2_N^−^,^[24]^ while for C_3_N^−^ only linear constitutional isomers have been considered.^[53]^ The hydrogen molecule H_2_ is weakly bound to the C_*n*_N^−^ anion and considered as a messenger molecule or *tag*. In this work, we distinguish between the (H_2_)C_*n*_N^−^ (C‐side tagged) and C_*n*_N^−^(H_2_) (N‐side tagged) conformers.

For the C_*n*_N^−^(H_2_) and (H_2_)C_*n*_N^−^ complexes with n=1-3 , we performed geometry optimizations within the explicitly correlated coupled cluster ansatz[^54^, ^55^] using a triple zeta basis,[^56^, ^57^] in brief CCSD(T)‐F12/cc‐pVTZ−F12. Compared to conventional coupled cluster theory, the F12 ansatz is known for faster basis set convergence so that our calculations may be best compared to CCSD(T) with a quintuple zeta basis, while maintaining reduced computational cost. On the same level of theory, we calculated the harmonic frequencies, dissociation energies as well as the thermochemistry within the rigid‐rotor harmonic oscillator approximation. The results are shown in the supplementary information. In accordance with Refs. [33,51,58], our calculations suggest that H_2_ tagging on the nitrogen side is favored for the CN^−^ species, while for the larger species, carbon‐side tagging is favored.

Anharmonic calculations were performed to improve upon the harmonic frequencies and to aid the interpretation of combination bands and/or overtones. In this respect, we calculated multi‐mode PES expansions[^59^, ^60^] with up to 3‐mode couplings using CCSD(T)‐F12/cc‐pVTZ−F12 level of electronic structure theory. The multi‐mode PES expansion needs multiple single point calculations on a grid, hence, we rely on the CCSD(T)‐F12 ansatz with a triple zeta basis for both C_2_N^−^ and C_3_N^−^, which has been shown to maintain a good reasonable compromise of computational time and accuracy in the calculation of the PES.^[61]^ Our choice of electronic structure theory is comparable to the CCSD(T) ansatz with a quadruple zeta basis chosen by Kołos et al.^[28]^ in their calculation of a cubic force field of C_3_N^−^. Note that the composite quartic force field for C_2_N^−^ computed by Rocha and Linnartz^[24]^ is also based on CCSD(T) level of theory, however, they include core‐correlation, scalar relativistic contributions and estimation of higher‐order electron correlation. Based on our multi‐mode PES, we calculated the vibrational structure by configuration averaged vibrational self‐consistent field (CAVSCF)^[62]^ and subsequent vibrational configuration interaction (VCI)[^44^, ^63^] with up to quadruple excitations (VCISDTQ). All calculations were performed using the Molpro 2022 software package.^[64]^

In terms of notation, we use two types of labels: (1) The normal mode description, for instance, $\delta_{\mathrm{CCN}}$ for the CCN bending notion and $\nu_{\mathrm{CC}-N}$ for CC−N stretching vibration. A visualization of the modes can be found in Figure S2 and S3. (2) The state identity description with the labels $v\;\nu_{i}^{l}$ , where we use the subscript *i* as previously introduced in literature.[^23^, ^24^] The prefix *v* denotes the vibrational quantum number and the superscript *l* denotes the absolute l‐type doubling quantum number. Additionally, these labels comprise the irreducible representation in brackets.

## Supporting Information

Additional references cited within the Supporting Information.[^65^, ^66^]

## Conflict of interest

The authors report there are no competing interests to declare.

1

## Supporting information

As a service to our authors and readers, this journal provides supporting information supplied by the authors. Such materials are peer reviewed and may be re‐organized for online delivery, but are not copy‐edited or typeset. Technical support issues arising from supporting information (other than missing files) should be addressed to the authors.

Supporting Information

## Data Availability

The used geometries for calculation, scripts and raw data are available at https://doi.org/10.5281/zenodo.7965386.
