# Supplementary material for: Vibrational Predissociation Spectra of C2N− and C3N−: Bending and Stretching Vibrations
Source: Chemphyschem. 2023 Jul 6;24(15):e202300262. doi: 10.1002/cphc.202300262 (PMC10962567; doi:10.1002/cphc.202300262)
Supplement: Supplementary file 1 — Supporting Information [file CPHC-24-0-s001.pdf]

ChemPhysChem

Supporting Information

Vibrational Predissociation Spectra of C_2N^- and C_3N^-: Bending and Stretching Vibrations

Franziska Dahlmann, Dennis F. Dinu, Pavol Jusko, Christine Lochmann, Thomas Gstir, Aravindh N. Marimuthu, Klaus R. Liedl, Sandra Brünken, and Roland Wester*

Single tagged vs. Double tagged spectrum

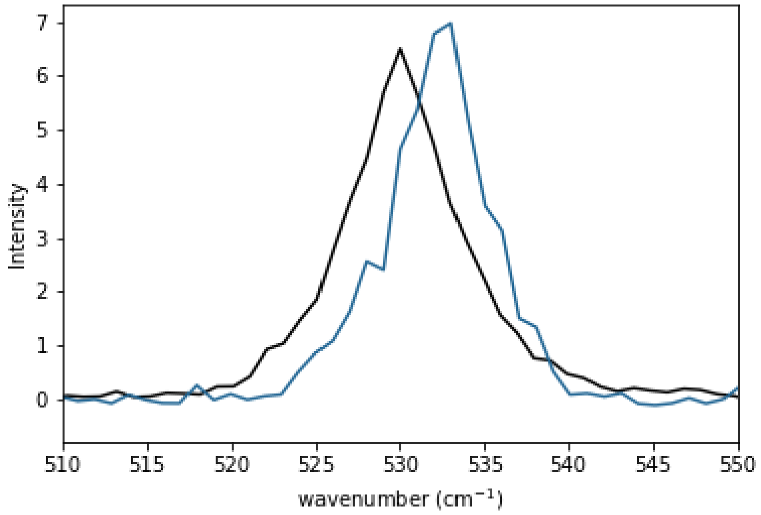

FIG. S1: Experimental vibrational infrared predissociation spectra of $C_3N^-(H_2)$ recorded at 15 K trap temperature in black and of $C_3N^-(H_2)_2$ in blue. The double tag spectrum shifts 3 cm^{-1} to higher wavenumbers.

Normal modes in $C_2N^-$and C_3N^-

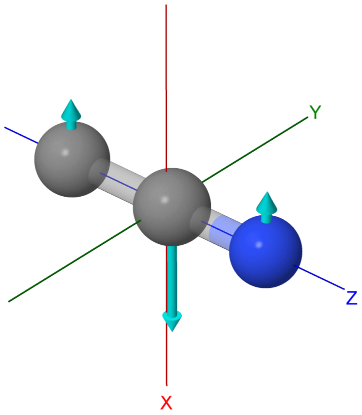

(a) δCCN $= \nu_2 =$ CCN bending mode

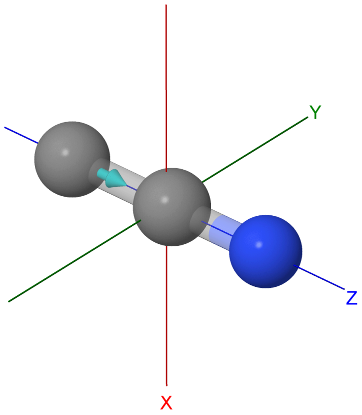

(b) νC-CN $= \nu_3 =$ C-CN stretching mode

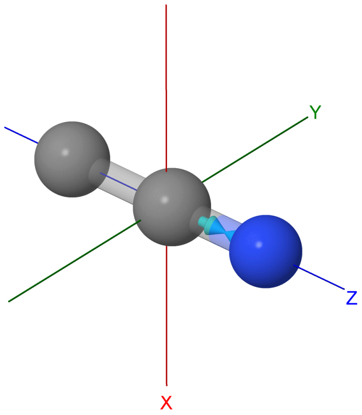

(c) νCC-N $= \nu_1 =$ CC-N stretching mode

FIG. S2: Normal vibrational modes of C_2N^-.

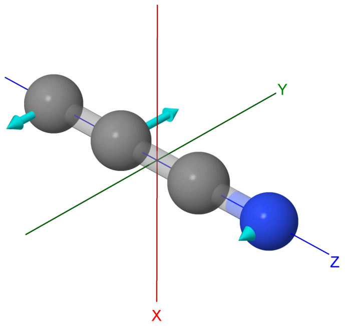

(a) δCCC = ν_5 = CCC bending mode

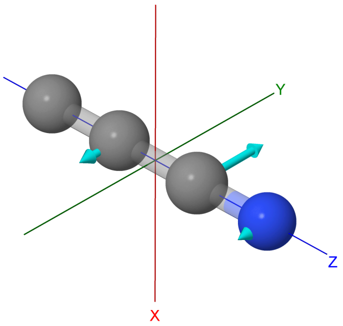

(b) δCCN = ν_4 = CCN bending mode

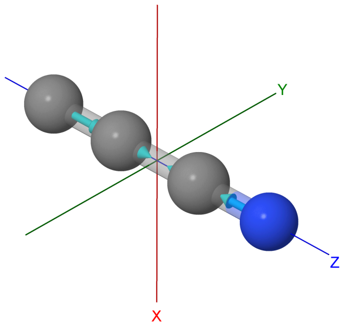

(c) νCC-CN = ν_3 = CC-CN stretching mode

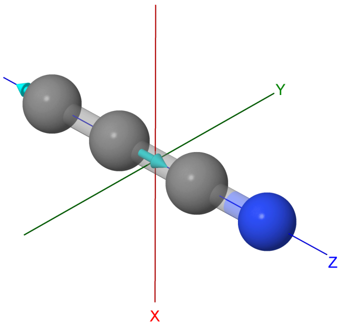

(d) νC-CCN = ν_2 = C-CCN stretching mode

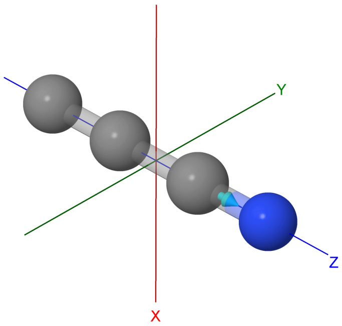

(e) νCCC-N = ν_1 = CCC-N stretching mode

FIG. S3: Normal modes of C_3N^-.

Tables with additional harmonic and anharmonic values

TABLE S1: Experimental frequencies of $C_2N^-(H_2)$ and anharmonic and harmonic frequencies of the bare C_2N^- anion. Positions are given in cm^{-1}, intensities in $km{\cdot}mol^{-1}$ (theo.) and relative intensity (exp.).

Mode	State	IRPD[a] freq./Int.	VCI (QFF)[b]	VCI (3D)[b] freq./Int.	VAR[c]	Harm.[d]	Harm.[e]
$\nu_{\mathrm{CC-N}}$	ν_1 (A1)	1698(1)/3.6	1700	1708/236.5	1696.5	1749	1753
$\nu_{\mathrm{C-CN}}$	ν_3 (A1)		1056	1058/1.3	1045.8	1116	1067
δ_{CCN}	ν_2 (E1)	454(1)/1.2	450	452/28.4	452.1	465	456
$2\nu_{\mathrm{CC-N}}$	$2\nu_1$ (A1)		3366	3393/73.4			3505
$2\nu_{\mathrm{C-CN}}$	$2\nu_3$ (A1)		2092	2100/0.5			2134
$2\delta_{\mathrm{CCN}}$	$2\nu_2$ (A1)		890	893/3.5			912
$\nu_{\mathrm{C-CN}}+\nu_{\mathrm{CC-N}}$	$\nu_3+\nu_1$			2765/28.1			2820
$\delta_{\mathrm{CCN}}+\nu_{\mathrm{CC-N}}$	$\nu_2+\nu_1$			2152/1.8			2209
$\delta_{\mathrm{CCN}}+\nu_{\mathrm{C-CN}}$	$\nu_2+\nu_3$			1516/0.1			1523

[a] This work: Infrared predissociation on $C_2N^-(H_2)$, [b] This work: Vibrational configuration interaction (VCI) calculations on a quartic force field (QFF) or on a multi-mode PES with up to 3-mode couplings (3D), in both cases at the CCSD(T)-F12 level of theory, [c] Ref. [24]: Variational (VAR) solution of the three-atom Hamiltonian on a composite quartic force field at the CCSD(T) level of theory, [d] Ref [23]: Harmonic calculations B3LYP/aug-cc-pVTZ level of theory, [e] This work: Harmonic calculations performed on the CCSD(T)-F12/cc-pVTZ-F12 level of theory.

TABLE S2: Experimental frequencies of $C_3N^-(H_2)$, SEVI, anharmonic and harmonic frequencies of the bare C_3N^- anion. Positions are given in cm^{-1}, intensities in $km{\cdot}mol^{-1}$ (theo.) and relative intensity (exp.).

Mode	State	IRPD freq./Int.	SEVI[c]	VCI (3D) [d] freq./Int.	Anharm.[e] freq./Int.	Anharm.[f] freq./Int.	Harm.[g]	Harm.[h]
ν_{CCC-N}	ν_1 (A1)	2180[a]		2178/804	2182.3/476	2182.3/476	2217	2210.3
ν_{C-CCN}	ν_2 (A1)	1952[a]		1939/62	1940.9/46	1940.9/46	1971	1964.8
ν_{CC-CN}	ν_3 (A1)	866(1)/3.1[b]		864/8	866.7/10	866.7/10	878	875.6
δ_{CCN}	ν_4 (A1)	530(1)/5.8[b]	538±8	527/0			530	532.8
δ_{CCC}	ν_5 (A1)		208±8	200/6			199	203.0
$2\nu_{CCC-N}$	$2\nu_1$ (A1)			4336/1			4435	
$2\nu_{C-CCN}$	$2\nu_2$ (A1)			3865/2			3943	
$2\nu_{CC-CN}$	$2\nu_3$ (A1)			1722/2			1755	
$2\delta_{CCN}$	$2\nu_4$ (E1)	1068(1)/0.6[b]		1063/3			1059	
$2\delta_{CCC}$	$2\nu_5$ (E1)	407(1)/0.3[b]		406/1			398	
$\nu_{C-CCN}+\nu_{CCC-N}$	$\nu_2+\nu_1$			4103/1			4189	
$\nu_{CC-CN}+\nu_{CCC-N}$	$\nu_3+\nu_1$			3041/13			3095	
$\delta_{CCN}+\nu_{CCC-N}$	$\nu_4+\nu_1$			2803/2			2849	
$\delta_{CCC}+\nu_{CCC-N}$	$\nu_4+\nu_1$			2698/0			2747	
$\nu_{CC-CN}+\nu_{C-CCN}$	$\nu_3+\nu_2$			2468/1			2501	
$\delta_{CCN}+\nu_{C-CCN}$	$\nu_4+\nu_2$			2375/0			2417	
$\delta_{CCC}+\nu_{C-CCN}$	$\nu_5+\nu_2$			2135/0			2171	
$\delta_{CCN}+\nu_{CC-CN}$	$\nu_4+\nu_3$			1386/0			1407	
$\delta_{CCC}+\nu_{CC-CN}$	$\nu_5+\nu_3$			1071/0			1077	
$\delta_{CCC}+\delta_{CCN}$	$\nu_5+\nu_4$			738/0			729	

[a] Ref. [33]: (IRPD) of $C_3N^-(D_2)$, [b] This work: IRPD of $C_3N^-(H_2)$, [c] Ref. [32]: slow electron velocity-map imaging (SEVI) of C_3N^-, [d] This work: Vibrational configuration interaction (VCI) based on a multi-mode PES with up to 3-mode couplings at the CCSD(T)-F12 level of theory, [e] Ref. [28]: Vibrational perturbation theory (VPT2) based on a cubic force field at the CCSD(T) level of theory, [f] Ref. [28]: Anharmonic frequency calculations CCSD(T)/aug-cc-pVQZ, [g] This work: Harmonic frequency calculations CCSD(T)-F12/cc-pVTZ-F12, [h] Ref. [28]: Harmonic vibrational calculations on the CCSD(T)/aug-cc-pVQZ level of theory.

Dissociation Energies and Conformers

Our theoretical CCSD(T)-F12/cc-pVTZ-F12 results show that (1) for the CN^-, the N-side tagging is favored, while for the $C_2N^-$and $C_3N^-$the C-side tagging is favored, and (2) the tagging, in general, becomes less favorable with larger carbon chain lengths. The calculated electronic dissociation energies D_e and their zero-point energy corrected counterparts D_0 are shown in Table S3. The $CN^-(H_2)$ conformer is the lower energy form of the complex, being 101 cm^{-1} more stable than the $(H_2)CN^-$conformer, in agreement with CCSD(T)-F12b/AV5Z calculations (128 cm^{-1})[42] and in agreement with the calculated well-depth difference of 124 cm^{-1} on the PES [65]. For longer-chain carbon chains, the preferred side of the tag changes so that the $(H_2)C_2N^-$ conformer is the lower energy form of the complex, being 35 cm^{-1} more stable than the $C_2N^-(H_2)$ conformer. The trend is continued with the $(H_2)C_3N^-$ conformer being 73 cm^{-1} more stable than the $C_3N^-(H_2)$ conformer. This trend is supported by previous studies [33, 51, 58]. In the following discussion, we thus focus on the lower-lying $(H_2)C_2N^-$ and $(H_2)C_3N^-$ conformer since the $C_2N^-(H_2)$ and $C_3N^-(H_2)$ conformers will only be formed in negligible amounts at the cold temperatures in the cryogenic ion trap.

TABLE S3: Electronic D_e and harmonic zero-point corrected D_0. Calculated at CCSD(T)-F12/cc-pVTZ-F12 level of theory.

	D_e / cm^{-1}		D_0^{Harm} / cm^{-1}	
n	$(H_2)C_nN^-$	$C_nN^-(H_2)$	$(H_2)C_nN^-$	$C_nN^-(H_2)$
1	712	838	147	248
2	723	669	196	161
3	663	537	138	65

As the electronic dissociation energies do not consider enthalpy and entropy contributions, statements (1) and (2) might not be validly compared to the experiment. Hence, we calculated the thermodynamics for the dissociation reaction using the KiSTheIP software, version 2021 [66]. In this approach, the species are treated as rigid rotor harmonic oscillators (RRHO) for an approximate evaluation of their partition functions and subsequent calculation of enthalpy and entropy through statistical thermodynamics. The thermodyamic

calculations show that the Nitrogen tagged complex $CN^-(H_2)$ is thermodynamically more stable than the Carbon side tagged one $(H_2)CN^-$. For C_2N^- and C_3N^- the tag to be on the Carbon side is favored. These results are in agreement with the statement (2) made for the electronic dissociation energies D_e and D_0^{Harm} (see Figure S4).

TABLE S4: Harmonic frequencies of hydrogen tagged C_nN^- (in cm^{-1}). Calculated at CCSD(T)-F12/cc-pVTZ-F12 level of theory.

Label	(H_2)*	C_2N^-	*(H_2)	(H_2)*	C_3N^-	*(H_2)
$\nu_{\mathrm{H-H}}$	4248		4294	4268		4328
$\nu_{\mathrm{C-N}}$	1752	1753	1755	2220	2217	2218
$\nu_{\mathrm{C-CCN}}$				1975	1971	1972
$\nu_{\mathrm{C-CN}}$	1069	1067	1069			
$\nu_{\mathrm{CC-CN}}$				879	878	881
δ^*_{CCN}	457	456	453	529	530	530
τH_2^*	508		461	498		417
δ_{CCC}				200	199	198
ΔH_2-C_nN	189		203	180		183
τC_nN	23		29	23		21

* In the harmonic approach, the δ_{CCN} and τH_2 modes in the tagged system show overlapping characteristics, making it difficult to distinguish between the two.

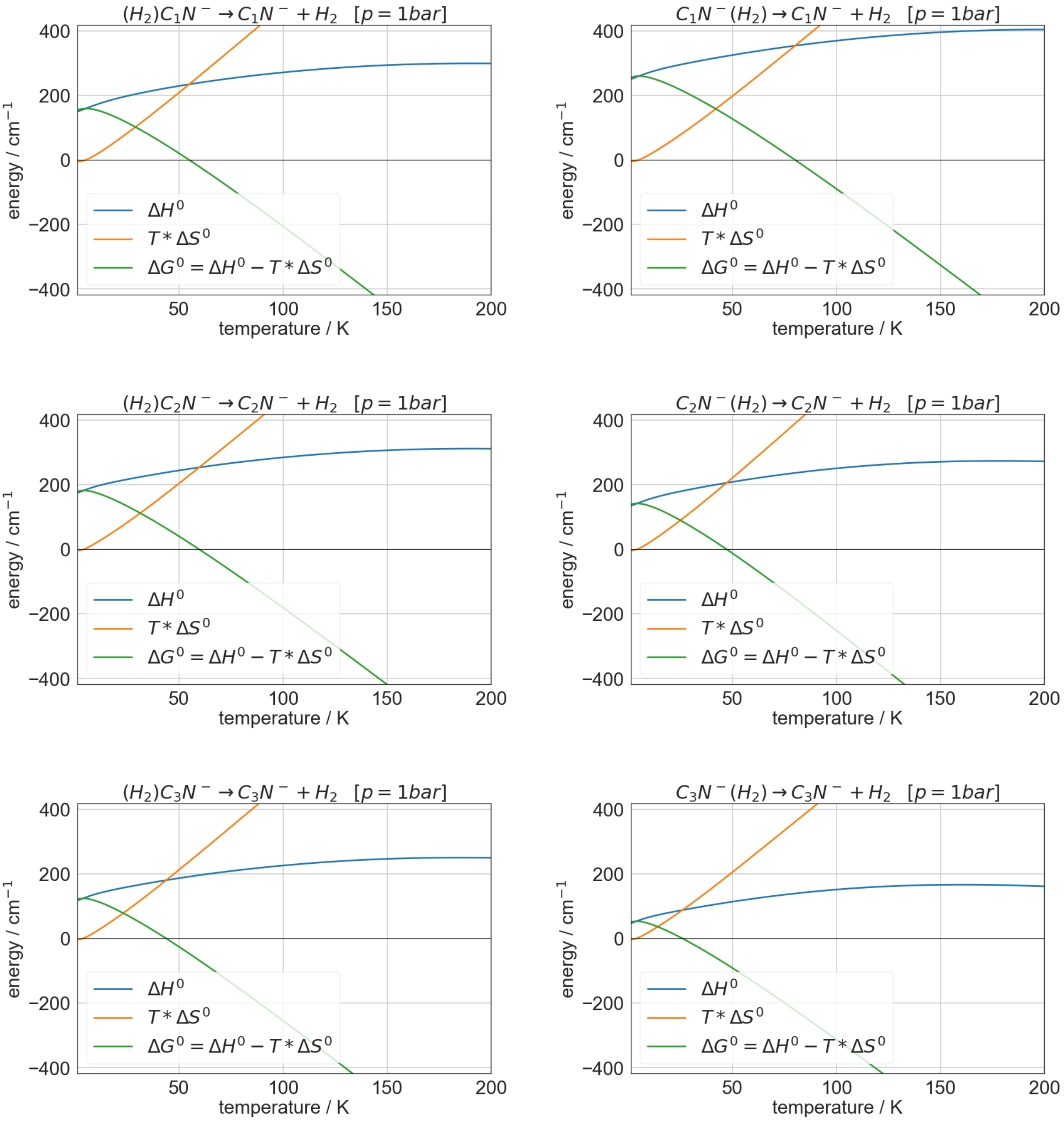

FIG. S4: Dissociation enthalpy ΔH^0, entropy ΔS^0 and free energy ΔG^0 for temperatures in between 0 K - 200 K and a pressure of 1 bar. Calculated within the RRHO approximation using the KiSTheIP software with electronic energies and harmonic frequencies at CCSD(T)-F12/cc-pVTZ-F12 level of theory using the Molpro software.
